# Epidemiological study of cutaneous leishmaniasis in Saesie Tsaeda-emba district, eastern Tigray, northern Ethiopia

**DOI:** 10.1186/s13071-015-0758-9

**Published:** 2015-03-07

**Authors:** Abrha Bsrat, Nega Berhe, Meshesha Balkew, Mekonnen Yohannes, Tsigemariam Teklu, Endalamaw Gadisa, Girmay Medhin, Adugna Abera

**Affiliations:** College of Veterinary Medicine, Mekelle University, P O Box 231, Mekelle, Ethiopia; Aklilu Lemma Institution of Pathobiology, Addis Ababa University College of Health Sciences, P O Box 1176, Addis Ababa, Ethiopia; Mekelle University College of Health Sciences, P O Box 231, Mekelle, Ethiopia; Armauer Hansen Research Institute, Addis Ababa, Ethiopia; Tigray Regional Health Bureau, Mekelle, Ethiopia

**Keywords:** Clinical assessment, Culture, Cutaneous leishmaniasis, Households, Sandfly, Saesie Tsaeda-emba, Smear

## Abstract

**Background:**

Cutaneous leishmaniasis (CL) is one of the endemic and neglected diseases known to exist in Ethiopian highlands. However, little is known about its epidemiological characteristics. Hence, this study was initiated and conducted from November 2011 to April 2012 to assess the epidemiological situation of CL in Saesie Tsaeda-emba District.

**Methods:**

A cross sectional design was employed in six randomly selected Peasant associations and a house to house survey was carried out in the District. Detailed clinical assessment, and smear and culture for *Leishmania* parasite detection were done to confirm clinical suspension. Polymerase Chain Reaction and Restriction Fragment Length Polymorphism (PCR-RFLP) analysis of the ribosomal DNA Internal Transcribed Spacer (ITS-1) sequences was used to type isolates. Sandfly collection was also conducted in possible micro-habitats of the target areas.

**Results:**

The overall prevalence of CL in the District was 14.0% (6.7% for active lesion and 7.3% for scar) with the highest prevalence amongst the age group of 10–19 years. Field isolates typed were *L. aethiopica.* Environmental and host risk factors significantly associated with CL distribution were age, study Peasant associations, presence of cave/gorge, walls with cracks and/or holes, presence of hyrax, animal burrow, animal dung and farm land near to residents’ houses. Five phlebotomine sandflies, *Phlebotomus longipes, Sergentomyia bedfordi, S.africana, S.schwetzi* and *S.antenata* were captured.

**Conclusion:**

All the precipitating factors in the area are indicative of the public health importance of CL although there has been little attention given. The present study is a starter for wider investigation into the mode of its transmission, incrimination of sandfly vectors and possible animal reservoirs. Detailed information will be the basis to launch effective control of CL in the area.

**Electronic supplementary material:**

The online version of this article (doi:10.1186/s13071-015-0758-9) contains supplementary material, which is available to authorized users.

## Background

In terms of global burden of the disease, leishmaniases represent the third most important vector-borne disease [1]. Due to their potentially disfiguring effects, Cutaneous (CL) and mucocutaneous Leishmaniasis (MCL) have significant social impact. However, since the disease is non-fatal, the attention given to prevention and control has been comparatively poor [2]. Ethiopia is located in North-Eastern Africa, occupying a total area of 1.14 million km2. The country is burdened with low human and economic development, serious environmental problems such as deforestation, overgrazing, soil erosion, desertification and high vulnerability to a changing climate. Forests in general have shrunk from 65% covering of the country and 90% of the highlands to 2.2% and 5.6% respectively [3]. Such changes are undoubtedly influencing the profile of vector-borne diseases [4,5].

Tigray Regional State is in Northern Ethiopia and covers a total area of about 80,000 km2, 65% of which is cultivated. It can be broadly divided into a number of highland blocks separated by deep river valleys. From an agro-ecological perspective it is characterized by sparse and irregular rainfall, and is highly drought-prone. The rich geographical diversity implies that certain ecological zones are confined to small areas, with human communities, the flora and the fauna highly adapted to subsist within them.

Saesie Tsaeda-emba District situated between 14° 11′ 14″ N latitude and 39° 33′ 50″ E longitude is located in eastern Zone of Tigray National Regional State at the north eastern edge of the Ethiopian highlands 976 km north of Addis Ababa (capital of the country).

CL is a disfiguring protozoan disease, with the potential of long term psychological and social consequences, especially in young women. In Ethiopia, CL is principally caused by *L. aethiopica* and it is widespread in the highland areas [6-8]. Though rarely, *L. tropica* and *L. major* have also been implicated in the lowland regions [9,10]. It is transmitted by several species of phlebotomine sandflies with *Phlebotomus longipes* and *P. pedifer* commonly identified as proven vectors from different parts of the county. Reservoir hosts so far known in Ethiopia are rock hyraxes, *Procavia capensis* and *Hetrohyrax brucei* for *L. aethiopica* [7] and the rodent, *Arvicanthis niloticus* for *L. major* [11]. In spite of reports on localized cutaneous leishmaniasis, mucocutaneous leishmaniasis and diffused cutaneous leishmaniasis, detailed information regarding its magnitude and epidemiology in the country is incomplete [12]. A study on CL in Saesie Tsaeda-emba District was therefore initiated and conducted to appreciate the magnitude of CL and identify major potential determinants of the disease which in turn assist in devising baseline information to develop national-wide CL information to control and prevent the disease.

## Methods

The study was conducted from November 2011 to April 2012 in Saesie Tsaeda-emba District located in eastern Zone of Tigray National Regional State at the north eastern edge of the Ethiopian highlands 976 km north of Addis Ababa (capital of the country) situated between 14° 11′ 14″ N latitude and 39° 33′ 50″ E longitude. Saesie Tsaeda-emba District is one of the 9 Districts in the Zone comprising 27 Peasant associations. The District has a total population of 153,003 (48.5%, 51.5% male and female respectively) and 35,179 households resulting in an average of 4.35 persons to a household (Saesie Tsaeda-emba District Health Bureau, 2011, unpublished). Of the total population, 86.4% are rural habitants. With an area of 2,511.47 square kilometers, Saesie Tsaeda-emba has a population density of 60.92 per km^2^ [13].

Altitude of the District ranges from 2357 to 3000 m.a.s.l. of which 96% belongs to Dega (highland) and has a semi-arid climate with annual rainfall and temperature of 350–500 mm and 13-20°C respectively. The predominantly unimodal rainfall (June to September) is characterized by high temporal and spatial variability. Severity of soil erosion in the area is a result of the mountainous and hilly topography, torrential rainfall, and low degree of vegetation cover (BoFED, 2007, unpublished).

A total of 2106 (979 males and 1,127 females) habitants from six peasant associations studies namely: *Edaga-hamus* (384)*, Emba-mezewle* (321)*, Hadush-hiwot* (467)*, Saesie* (262)*, Geblen* (324) *and Emba-asmena* (348) from 433 households were selected in the District using multistage simple random sampling strategy. Participants were categorised into four age groups (0–9, 520; 10–19, 499; 20–29, 386; and ≥30, 701). All selected households were visited and household heads were approached to collect the required information using a pre-tested questionnaire. All registered CL cases (lesions/scars) during house-to-house survey were subjected to detailed clinical examination taking the number of individuals with active lesions or scars due to CL, number of lesions/scars, site, size, morphology and type of lesion into account [14,15]. Parasitological confirmation was completed based on smear and culturing on Novy MacNeal Nicolle (NNN) Media of skin scrapings. Typing of the *Leishmania* species was also achieved by PCR amplification and RFLP analysis of the ribosomal DNA Internal Transcribed Spacer (ITS) sequences [16]. Furthermore, detailed observation on the nearby micro-environment was examined for possible risk factors. Sandflies were collected from likely resting habitats (outdoor and indoor micro-habitats) using sticky A4 size paper sheets coated with motor oil and CDC miniature light traps [17]. Each collected sandfly was then sorted by sex and genera, and identified to species level using morphological keys [18,19].

### Ethical approval

Ethical approval was obtained from the Institutional Review Board (IRB) of ALIPB and the Tigray Science and Technology Agency represented by College of Health Sciences of Mekelle University. Permission from the Tigray regional health bureau and respective district authorities were obtained subsequently. Verbal informed consent was obtained from the head of the households selected after explanation of the purpose of the study in Tigrigna. Signed consent of those with active lesion was obtained from individual cases or family/guardians.

## Results

### Prevalence of CL

Of the population surveyed (2106), 14% showed clinical evidence of CL infection nearly in equal proportion of active CL and cases with scars (Table 1, Additional file 1). All the active lesions observed during the study were localized type (LCL).

**Table 1:** **Prevalence of active lesion & scar among study groups in Saesie Tsaeda-emba District (Nov.2011-Apr.2012)**

**Peasant associations**	**CL infection status**	**Age & Sex**								**Total**	
		**0 – 9 yrs**		**10 – 19 yrs**		**20 – 29 yrs**		**≥30 yrs**			
		**M**	**F**	**M**	**F**	**M**	**F**	**M**	**F**	**M**	**F**
**Edaga-hamus**	CL active lesion	1	4	4	4	0	2	2	0	7	10
	CL scar	0	1	4	6	6	4	1	3	11	14
**Emba-mezewle**	CL active lesion	1	2	2	0	0	0	2	3	5	5
	CL scar	4	2	6	7	7	4	4	6	21	19
**Hadush-hiwot**	CL active lesion	7	5	6	6	0	1	2	1	15	13
	CL scar	2	2	7	9	4	8	6	6	19	25
**Saesie**	CL active lesion	2	2	3	5	0	0	0	1	5	8
	CL scar	3	1	3	1	0	3	3	0	9	5
**Geblen**	CL active lesion	2	0	4	3	2	2	2	1	10	6
	CL scar	0	0	3	2	0	5	2	1	5	8
**Emba-asmena**	CL active lesion	15	10	16	11	0	1	1	3	32	25
	CL scar	0	1	4	4	0	1	5	3	9	9
**Total**	**CL active lesion**	**28**	**23**	**35**	**29**	**2**	**6**	**9**	**9**	**141 (6.7%)**	
	**CL scar**	**9**	**7**	**27**	**29**	**17**	**25**	**21**	**19**	**154 (7.3%)**	

M = male; F = female; yrs = years.

During this study, highest age specific active lesion prevalence was noted in the age group 10–19 (12.8%; 64/499) followed by age group of 0–9 (9.8%; 51/520). Considering gender distribution, relatively higher CL proportion was determined in males (7.6%; 74/979) rather than females (5.9%; 67/1,127). Furthermore, the prevalence of CL lesions/scars showed marked variation between the Peasant associations surveyed with the highest overall prevalence of CL lesions/scars in Emba-asmena Peasant association (Table 2).

**Table 2:** **Distribution of CL among age and sex groups, and Peasant associations in Saesie Tsaeda-emba District (Nov.2011-Apr.2012)**

**Categories**		**Active lesion (%)**	**Scar (%)**	**Total CL + ve (%)**
**Age**	0-9	51 (9.8)	16 (3.1)	67 (12.9)
	10-19	64 (12.8)	56 (11.2)	120 (24.0)
	20-29	8 (2.1)	44 (10.9)	50 (13.0)
	≥30	18 (2.6)	40 (5.7)	58 (8.3)
**Sex**	Male	74 (7.6)	74 (7.6)	148 (15.2)
	Female	67 (5.9)	80 (7.1)	147 (13.0)
**Peasant associations**	Edaga-hamus	17 (4.4)	25 (6.5)	42 (10.9)
	Emba-mezewle	10 (3.1)	40 (12.5)	50 (15.6)
	Hadush-hiwot	28 (6.0)	44 (9.4)	72 (15.4)
	Saesie	13 (5.0)	14 (5.3)	27 (10.3)
	Geblen	16 (5.0)	13 (4.0)	29 (9.0)
	Emba-asmena	57 (16.4)	18 (5.2)	75 (21.6)

+ve = positive, −ve = negative.

Active lesions and scars of CL cases were recorded on face, upper (hands) and lower (legs) extremities although their distribution in different body parts is variable (Table 3). The majority of active lesions were observed on the face in which the cheeks and the nose were most affected. A similar pattern was observed with the distribution of scars on different parts of the body.

**Table 3:** **Distribution of active lesions and scars of CL cases on body parts from Saesie Tsaeda-emba District (Nov.2011-Apr.2012)**

**Location**	**Active lesions (%)**	**Scars (%)**	Total* (%)
**Cheek**	62 (37.1)	70 (42.7)	132 (39.9)
**Nose**	45 (26.9)	39 (23.8)	84 (25.4)
**Hand**	22 (13.2)	18 (11.0)	40 (12.1)
**Forehead**	14 (8.4)	14 (8.5)	28 (8.5)
**Ear**	6 (3.6)	5 (3.0)	11 (3.3)
**Lip**	6 (3.6)	6 (3.7)	12 (3.6)
**Chin**	4 (2.4)	2 (1.2)	6 (1.8)
**Neck**	2 (1.2)	2 (1.2)	4 (1.2)
**Leg**	6 (3.6)	7 (4.3)	13 (4.0)
**Others**	0 (0.0)	1 (0.6)	1 (0.3)
**Total**	**167 (100)**	**164 (100)**	**331 (100)**

*Multiple lesions on a single individual on different location of body parts were counted with their respective locations.

Involvement of more than one anatomical region per individual was not uncommon although the proportion of cases with single active lesion and scars were 78.7% and 92.9% respectively. However, cases with three or more active lesions constituted 4.3% and 0.6% for active lesion and scars respectively of the total cases recorded (Table 4).

**Table 4:** **Number of active lesions and scars among patients with respect to their sex in Saesie Tsaeda-emba District (Nov.2011-Apr.2012)**

**Lesion No**	**Active lesion (%)**		**Total (%)**	**Scars (%)**		**Total (%)**
	**Male**	**Female**		**Male**	**Female**	
**One**	58 (41.1)	53 (37.6)	111(78.7)	67 (43.5)	76 (49.4)	143(92.9)
**Two**	13 (9.2)	11 (7.8)	24 (17.0)	7 (4.5)	3 (1.9)	10 (6.5)
**Three & more**	3 (2.1)	3 (2.1)	6 (4.3)	0 (0.0)	1 (0.6)	1(0.6)
**Total**	**74 (52.5)**	**67 (47.5)**	**141(100)**	**74 (48.1)**	**80 (41.9)**	**154(100)**

### Smear and culture results

Skin scrapings from a total of 43 participants who had active lesions were smear examined and amastigotes were visualized from 69.8% of them. Similarly, of the 31 samples of active lesions presented to culture 32.3% were found positive for CL (Table 5).

**Table 5:** **Summary of culture and smear results of CL cases from Saesie Tsaeda-emba District (Nov.2011-Apr.2012)**

**Categories**	**Positive (%)**	**Negative (%)**	**Contaminated (%)**	**Total (%)**
**Culture**	10 (32.3)	10 (32.3)	11 (35.5)	31 (100)
**Smear**	30 (69.8)	13 (30.2)	NA	43 (100)

NA = Not applicable.

### PCR Amplifications with the ITS-1 Primer Pair (LISTR/L5.8S) and RFLP Analysis

**Figure 1:**
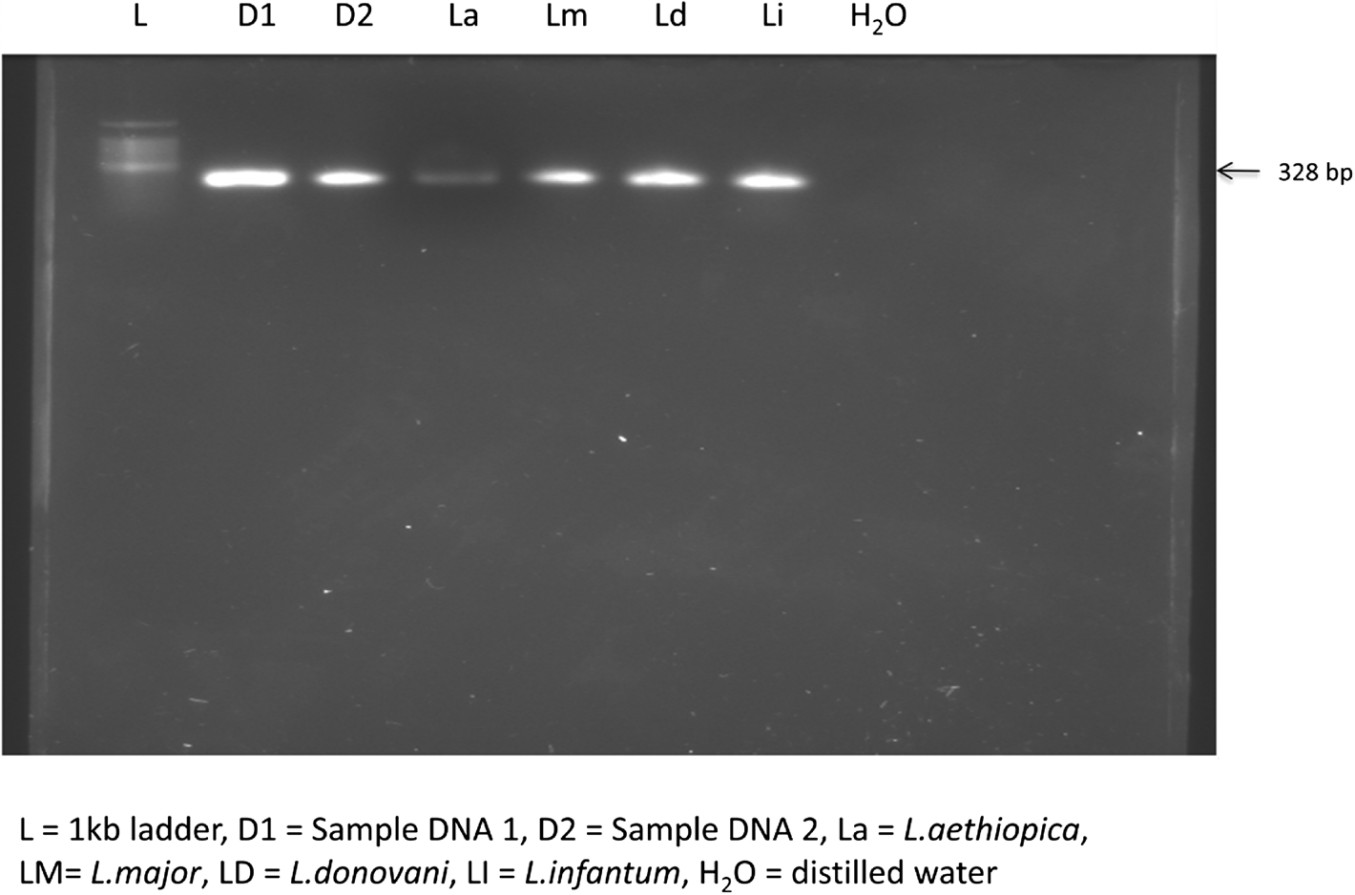
**PCR products of ITS-1 from promastigote DNA of field isolate and reference strains with primer pairs LISTR/L5.8S (Nov.2011-Apr.2012).**

**Figure 2:**
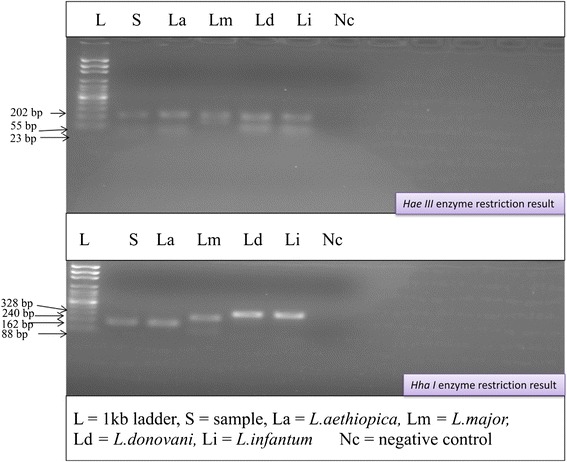
**PCR-ITS-1-RFLP of amplicons of field isolate and reference strain after digestion with** ***Hae III*** **and** ***Hha I*** **(Nov.2011-Apr.2012).**

The PCR of the ITS-1 with primer pairs LISTR/L5.8S gave approximately 328 bp product for both reference strains and field isolates from cultured promastigotes DNA of the study District (Figure 1). When the ITS-1 PCR product was digested by *Hae III, L.aethiopica* reference strains and field isolates produced three bands of size 202 bp, 55 bp and 23 bp (Figure 2). When the ITS-1 PCR product was digested by *Hha I, L. aethiopica* reference strain and the field isolates produced a band approximately 162 bp size. According to the sequence information from gene bank, the 162 bp is actually a superimposition of 164 bp and 162 bp bands (accession no AJ000311). Two bands of about 88 bp and 240 bp sizes were produced for *L. major* whereas, *L. infantum* and *L. donovan*i gave single band size of 328 bp showing that there is no restriction site in the ITS-1 region for these strains. Thus, the field isolates produced identical bands with *L. aethiopica* reference strain.

### Environmental and host factor analysis

For this study, environmental factors included presence of cave and/or gorge in 300 m radius from the participants house, presence of animal burrow in the compound, presence of animal dung in the compound, presence of hyrax near the participants house, wall condition of their house and presence of farm land within 300 m radius from their house were assessed to look at their impact with the distribution of CL (Table 6). As per recorded output, higher prevalence was recorded from those whose habitats were near to cave and/or gorge (15.7%), animal burrow (15.4%), animal dung (15.5%), hyrax shelter (17.3%), farm land (15.3%), and with house wall cracks/holes (14.7%).

**Table 6:** **Summary of environmental factors association with CL prevalence in Saesie Tsaeda-emba District (Nov.2011-Apr.2012)**

**Categories**		**CL + ve (%)**	**CL –ve (%)**
**Cave/gorge (300 m)**	Yes	205 (15.7)	1104 (84.3)
	No	90 (11.3)	707 (88.7)
**Hyrax near home (300 m)**	Yes	240 (17.3)	1144 (82.6)
	No	55 (7.6)	667 (92.4)
**Wall condition**	Cracked/holes	289 (14.7)	1671 (85.3)
	No crack/holes	6 (4.3)	140 (95.7)
**Animal burrow near home**	Yes	267 (15.4)	1463 (84.6)
	No	28 (7.4)	348 (92.6)
**Animal dung near home**	Yes	263 (15.5)	1439 (84.5)
	No	32 (7.9)	372 (92.1)
**Farm near home (300 m)**	Yes	258 (15.3)	1430 (84.7)
	No	37 (8.9)	381 (91.1)

+ve = positive, −ve = negative, X^2^ = Chi-square,

The crud odds ratios of the present study are presented in Table 7. Significant variation in the prevalence of CL among the different age groups, peasant associations, living in house with wall cracks/holes, and living near to caves/gorges, hyrax habitats, animal burrow, animal dung and farm land (Table 7). Individuals in the age group of 10–19 years were 2.14 (OR = 2.14; 95% CI: 1.54-2.97) and 1% (OR = 1.01; 95% CI: 0.67-1.48) times more likely to have CL compared to individuals in the age group of 0–9 years whereas those aged greater than 30 years were 39% (OR = 0.61; 95% CI: 0.42-0.88) less likely to be exposed by CL. Those participants who lived near to hyrax shelter were 2.54 times more likely to be infected by CL compared to individuals who live far from hyrax living area (OR = 2.54; 95% CI: 1.87-3.46). With similar concept habitants of Emba-asmena peasant association were found 2.24 (OR = 2.24; 95% CL: 1.48-3.37), Emba-mezewle peasant association 50% (OR = 1.50; 95% CL: 0.97-2.33) and Hadush-hiwot 48% (OR = 1.48; 95% CL: 0.99-2.23) times more likely to be exposed to CL. However, those habitants from Saesie peasant association were 6% (OR = 0.94; 95% CL: 0.56-1.55) and from Geblen peasant association were 20% (OR = 0.80; 95% CL: 0.49-1.32) less likely to be infected by CL than those who lived at Edaga-hamus peasant association. Furthermore, participants living near to caves/holes, animal burrow, animal dung and farm land were found 46% (OR = 1.46; 95% CI: 1.12-1.90), 2.27 (OR = 2.27; 95% CI: 1.51-3.41), 2.12(OR = 2.12; 95% CI: 1.44-3.12) and 86% (OR = 1.86; 95% CI: 1.29-2.67) times more likely to be infected by CL than those inhabitants living far away from caves/holes, animal burrow, animal dung and farm land respectively.

**Table 7:** **Univariate logistic regression analysis in assessing the effect of environmental and host factors on the odds of being positive for CL in Saesie Tsaeda-emba District (Nov.2011-Apr.2012)**

**Categories**		**Univariate**	
		**OR**	**CI (95%)**
**Age**	0-9	1	
	10-19	2.14	1.54 - 2.97
	20-29	1.01	0.67- 1.48
	≥30	0.61	0.42- 0.88
**Peasant associations**	Edaga-hamus	1	
	Emba-mezewle	1.50	0.97 - 2.33
	Hadush-hiwot	1.48	0.99 - 2.23
	Saesie	0.94	0.56 - 1.55
	Geblen	0.80	0.49 - 1.32
	Emba-asmena	2.24	1.48 - 3.37
**Cave/gorge (300 m)**	No	1	
	Yes	1.46	1.12 - 1.90
**Hyrax near home (300 m)**	No	1	
	Yes	2.54	1.87 -3.46
**Wall condition**	No crack/holes	1	
	Cracked/holes	4.04	1.77 - 9.22
**Animal burrow near home**	No	1	
	Yes	2.27	1.51 - 3.41
**Animal dung near home**	No	1	
	Yes	2.12	1.44 - 3.12
**Farm near home (300 m)**	No	1	
	Yes	1.86	1.29 - 2.67

**OR** = Odds Ratio, **CI** = Confidence Interval.

### Indigenous knowledge of respondents on CL

Indigenous knowledge of questionnaire respondents (household heads and CL suspected) showed the majority to identify the disease (Table 8). However, none of them know the causative agent, source and mode of transmission of the disease. Rather they believed CL to be a genetic disease and all were unaware of the disease’s association with hyraxes. With regards to treatment, 99% of CL suspected cases use traditional treatment. Other patients did not seek treatment. All patients have no knowledge of modern medical treatment. Various local treatments are in use, application of herbs and holly water “Tsebel” being the most common.

**Table 8:** **Summary of indigenous knowledge of respondents (household heads and CL cases) on CL in Saesie Tsaeda-emba District (Nov.2011-Apr.2012)**

**Questionnaire data**	**Responses**	
	**Yes (%)**	**No (%)**
**CL identification**	510 (82.7)	107 (17.3)
**Cause of CL**	0 (0)	617 (100)
**Source of CL**	0 (0)	617 (100)
**CL transmission mode**	0 (0)	617 (100)
**Relation of CL with hyrax**	0 (0)	617 (100)
**Modern treatment**	0 (0)	295 (100)
**Traditional treatment**	293 (99.3)	2 (0.7)

### Identification of phlebotomine sandflies in the study district

One hundred and six sandflies belonging to 2 genera, *Phlebotomus* and *Sergentomyia* were identified (Table 9). The overall species composition was one *Phlebotomus* species, *P.longipes* and four *Sergentomyia* species namely, *Sergentomyia bedfordi, S.africana, S.schwetzi* and *S.antenata. P.longipes* were collected from both indoor and outdoor methods. However, all *Sergentomyia* species were caught outdoors.

**Table 9:** **Description of identified phlebotomine sandflies in Saesie Tsaeda-emba District (Nov.2011-Apr.2012)**

**Trap types**	**species**	**Number**		**Total**
		**Male**	**Female**	
**CDC (indoor)**	*Phlebotomus longipes*	0	2	2
**Sticky (Outdoor)**	*P. longipes*	5	1	6
	*Sergentomyia bedfordi*	21	50	71
	*S.africana*	13	8	21
	*S.schwetzi*	5	0	5
	*S.antenata*	1	0	1
**Total**		45 (42.5%)	61 (57.5%)	106 (100%)

## Discussion

The overall prevalence of active CL lesions in Saesie Tsaeda-emba District is found to be higher than previously reported elsewhere in the country using similar methodology including Sebeta [20], Kutaber [7], Ocholo [21], Silti [22] and Tigray (Mekonnen and Zerihun, unpublished data). The variation might be due to different factors including heterogeneity to exposure (entomological, environmental or behavioral factors) (micro-ecological variation). Moreover, this study revealed the presence of a localized form of CL and shed some light on the magnitude and public health significance of the disease in the District. LCL is known to exist in similar settings in other parts of Ethiopia [6,15,23-25].

Age groups 10–19 and below were found to be the most affected by CL (Table 2), implying, younger individuals are at risk. This result agrees with the reports from Kutaber [7], Addis Ababa [15] and Silti [22]. However, this is different to reports from endemic areas where prevalence of CL is highest among children of age group 0–10 [7,21,26]. This may be attributed to host behavior (probability of exposure) as those individuals (most affected age groups) frequently visit the gorges and escarpments (containing the hyrax shelters) during the evenings for leisure and recreation or for fetching water and firewood. Many individuals from this age group keep crops from wild animals mainly during the rainy season near the escarpments and gorges. Others go to the churches, which are also commonly located at the escarpments and gorges and spend many hours in spiritual ceremonies held in the evenings and mornings, which may increase their chance of being bitten by sandflies. Furthermore, it might be due to the custom of mothers in Tigray to carry small babies on their backs, wrapped with a scarf, so the under-five age groups are completely covered and protected from sandfly bites when outdoors. Merely based on these observations, it is hardly possible to point out where and when sandflies bite humans. The presence of greater scars on elder individuals showed that the disease has existed in the District for longer periods with the determined associated environmental and host risk factors.

The prevalence of CL is found significantly variable among the study participants in the 6 study Peasant associations with higher CL prevalence among those living in Emba-asmena (Table 6). This variation could be associated with the existing micro-ecological risk factors. The Emba-asmena ecological situation seemed to be important for the potential dynamics of CL as it is surrounded by escarpments and rocky hills (full of caves, cracks and crevices), and Haimele and Sahawa gorges which favor the presence of rock hyraxes and provide an ideal place for the survival of sandflies compared to the other Peasant associations. In line with other reports [7,27,28] which indicated that infection rates varied significantly from place to place depending primarily on the proximity of hyrax colonies and vector habitats to human dwellings and activities (such as land use, firewood collection, herding domestic animals), and environmental factors including topography, wind direction and intensity in Ethiopia. Besides, transmission within population is limited to areas corresponding to the short flight range of sandflies and the reservoir distributions [29].

The isolates typed from the present study sites are *L. aethiopica*, with similar RFLP pattern as the *L. aethiopica* reference strain (Figures 1 and 2). This result provides additional evidence for *L. aethiopica* to be the principal aetiologic agent of CL in Ethiopian high lands including Tigray.

Hyraxes live in almost all banks of the gorges and/or caves in the escarpments of the study District. Univariate analysis indicates that individuals living closer to hyrax vicinity (300 meters) are at relatively higher risk than those living far away and statistically significant association was found (Table 7) indicating that the disease seems to be of zoonotic nature. This result is consistent to previous reports from Kutaber [7], Addis Ababa [15] and Silti [22]. This might be due to close association of sandflies and reservoir. Sandflies are poor flyers, usually fly quite low and will remain in the vicinity of their breeding and resting site. Hence, the probability of humans being bitten by sandflies is considerably affected by the distance of the reservoir host (or its dwelling place) from their house [26,29,30]. Furthermore, the intimate ecological association of rock hyraxes with CL is considered as a typical characteristic of the Ethiopian CL caused by *L. aethiopica* [6,7,15].

Although the majority of the questionnaire respondents (household heads and CL suspected) are capable of identifying CL from other skin diseases (Table 8), they could not be free from leishmania infection. This indicates that their indigenous knowledge is not protective from CL. This might be due to their lack of awareness on the scientific dynamicity of the disease. Besides, their wrong belief (CL is a genetic disease) this may increase CL burden in such a way that patients might be afraid of contacting health centers to obtain modern treatment but instead seek traditional treatments. Thus, this concept can have great impact on the higher prevalence of the disease with regard to its prevention by the population themselves.

The presence of sandflies was also observed in the burrows where hyraxes live and the compounds of the houses and *P.longipes* was collected from both indoor and outdoor locations (Table 9). *P.longipes* is the vector of CL in Ethiopian highlands [7,31] and therefore, it could be responsible for the transmission of CL in Saesie Tsaeda-emba District although the role of other sandflies could not be ruled out because of limited entomological study.

## Conclusions

As in other parts of the country, the study showed that this important disease has been neglected in the study area but it remains a challenging public health problem because of the various ideal environmental risk factors. The presence of numerous scars on elder individuals suggests that the disease has prevailed for several years. *L.aethiopica* is the causative agent of CL in the District similar to other places in the country. Those people settled close to hyrax habitats, animal burrows, animal dung disposal areas and poor housing conditions such as wall cracks and/or holes are more exposed to the disease. The importance of the risk factors identified in this study should be investigated further and detailed epidemiological studies should be conducted in the District to identify the mode of transmission, role of the identified sandfly species and to incriminate possible animal reservoirs.

## Additional file

Additional file 1:
**Study population.**
