# Supplementary material for: Epidemiological study of cutaneous leishmaniasis in Saesie Tsaeda-emba district, eastern Tigray, northern Ethiopia
Source: Parasit Vectors. 2015 Mar 7;8:149. doi: 10.1186/s13071-015-0758-9 (PMC4359476; doi:10.1186/s13071-015-0758-9)
Supplement: Additional file 1: — Study population. [file 13071_2015_758_MOESM1_ESM.doc]

Additional file 1. Study population

A total of 433 households were visited and 2,106 individuals (46.5% males and 53.5% females) were screened for CL in the six kebelles surveyed (mean: 4.9 persons per household) (Table 10).

**Table 10:** Sex and age distribution of study population in each selected Kebelles of Saesie Tsaeda-emba Wereda (Nov.2011-Apr.2012)

| **Kebelles** | **HHs** | **Individuals** | **Sex** | | **Age groups** | | | |
| --- | --- | --- | --- | --- | --- | --- | --- | --- |
|  |  |  | **Male** | **Female** | **0-9** | **10-19** | **20-29** | **≥30** |
| **Edaga-hamus** | 81 | **384** | 177 | 207 | 114 | 79 | 87 | **104** |
| **Emba-mezewle** | 60 | **321** | 150 | 171 | 94 | 71 | 52 | **104** |
| **Hadush-hiwot** | 86 | **467** | 213 | 254 | 101 | 107 | 86 | **173** |
| **Saesie** | 57 | **262** | 119 | 143 | 60 | 58 | 53 | **91** |
| **Geblen** | 50 | **324** | 152 | 172 | 57 | 93 | 60 | **114** |
| **Emba-asmena** | 99 | **348** | 168 | 180 | 94 | 91 | 48 | **115** |
| **Total** | **433** | **2106** | **979** | **1127** | **520** | **499** | **386** | **701** |

HHs = households
